# Supplementary material for: Life Cycle Assessment and Impact Correlation Analysis of Fly Ash Geopolymer Concrete
Source: Materials (Basel). 2021 Dec 1;14(23):7375. doi: 10.3390/ma14237375 (PMC8658180; doi:10.3390/ma14237375)
Supplement: Supplementary file 1 [file materials-14-07375-s001.zip › materials-1449583-supplementary.pdf]

materials

Article

Life Cycle Assessment and Impact Correlation Analysis of Fly Ash Geopolymer Concrete

Xiaoshuang Shi [1,*], Cong Zhang [2], Yongchen Liang [2], Jinqian Luo [2], Xiaoqi Wang [2], Ying Feng [2], Yanlin Li [2], Qingyuan Wang [1,2,3] and Abd El-Fatah Abomohra [4]

1 Key Laboratory of Deep Earth Science and Engineering (Ministry of Education), Department of Architecture and Environment, Sichuan University, Chengdu 610065, China; wangqy@scu.edu.cn
2 Failure Mechanics and Engineering Disaster Prevention and Mitigation Key Lab of Sichuan Province, Sichuan University, Chengdu 610065, China; zhangcong@stu.scu.edu.cn (C.Z.); liangyc@stu.scu.edu.cn (Yongchen Liang); luojinqian@stu.scu.edu.cn (J.L.); wangxqi0126@163.com (X.W.); phying@stu.scu.edu.cn (Y.F.); tliyanlin@163.com (Yanlin Li)
3 Department of Mechanical Engineering, Chengdu University, Chengdu 610106, China
4 Department of Environmental Engineering, School of Architecture and Civil Engineering, Chengdu University, Chengdu 610106, China; abomohra@cdu.edu.cn
* Correspondence: shixs@scu.edu.cn

Citation: Shi, X.; Zhang, C.; Liang, Y.; Luo, J.; Wang, X.; Feng, Y.; Li, Y.; Wang, Q.; Abomohra, A.E.-F. Life Cycle Assessment and Impact Correlation Analysis of Fly Ash Geopolymer Concrete. *Materials* **2021**, *14*, 7375. https://doi.org/10.3390/ma14237375

Academic Editors: Thomas N. Kerestedjian and Alexander Karamanov

Received: 20 October 2021
Accepted: 29 November 2021
Published: 1 December 2021

Publisher's Note: MDPI stays neutral with regard to jurisdictional claims in published maps and institutional affiliations.

Copyright: © 2021 by the authors. Licensee MDPI, Basel, Switzerland. This article is an open access article distributed under the terms and conditions of the Creative Commons Attribution (CC BY) license (http://creativecommons.org/licenses/by/4.0/).

Table S1. Characteristics of aggregates.

Material	Maximum diameter (mm)	Linear mean diameter (mm)	Stacking density (kg/m³)	Apparent density (kg/m³)
Coarse aggregates	22	12.38	1479	2632
Fine aggregates	4	0.33	1342	2381

Table S2. Characteristics and chemical components of class F fly ash.

Composition	SiO_2	Al_2O_3	CaO	Fe_2O_3	MgO	K_2O	P_2O_5	TiO_2	Na_2O
Quantity (%)	74.18	9.44	5.53	4.22	2.48	1.46	1.41	0.96	0.32

Table S3. Mix proportion of fly ash GPC under different strength grades (kg/m3).

Strength grades		C_{NaOH} (mol/L)	SS/SH	S/F	CA	S	FA	Water	NaOH	Na_2SiO_3
C40	Mix 1	8.00	2.00	0.40	1212.00	544.00	460.00	45.30	16.10	122.70
	Mix 2	8.00	2.50	0.44	1201.00	539.00	460.00	42.20	15.00	142.90
	Mix 3	10.00	4.00	0.48	1186.00	533.00	460.00	30.30	13.90	176.60
	Mix 4	10.00	3.00	0.52	1174.00	527.00	460.00	40.00	18.30	180.90
C60	Mix 5	12.00	1.50	0.38	1180.00	530.00	500.00	48.60	27.40	114.00
	Mix 6	14.00	4.00	0.40	1212.00	544.00	460.00	21.90	14.90	147.20
	Mix 7	12.00	2.50	0.41	1132.00	508.00	540.00	40.20	22.70	157.10
	Mix 8	12.00	2.50	0.52	1174.00	527.00	460.00	43.70	24.70	170.90
C70	Mix 9	12.00	1.50	0.35	1152.00	518.00	540.00	48.56	27.44	114.00
	Mix10	12.00	2.00	0.35	1152.00	518.00	540.00	40.47	22.86	126.67
	Mix11	14.00	3.00	0.44	1201.00	539.00	460.00	29.09	19.72	151.30
	Mix12	12.00	2.00	0.48	1187.00	533.00	460.00	46.86	26.47	146.67

[1] CNaOH= NaOH concentration.
[2] SS/SH= Sodium silicate solution: Sodium hydroxide.
[3] S/F= Alkali activator solution: Fly ash.
[4] CA= Coarse aggregates，S= Fine aggregates.
[5] FA= Fly ash.

Table S4. Mix proportion of OPC concrete under different strength grades (kg/m3).

Strength grades		CA	S	Cement	Water	Concrete reducing water agent
C40	Mix 1	1215.00	572.00	415.00	166.00	0.00
	Mix 2	1104.00	736.00	440.00	170.00	0.00
	Mix 3	1301.00	481.00	513.00	205.00	0.35
	Mix 4	1301.00	481.00	513.00	205.00	0.40
C60	Mix 5	1198.00	617.00	486.00	170.00	4.90
	Mix 6	1151.00	647.00	494.00	158.00	4.94
	Mix 7	1044.00	696.00	500.00	160.00	5.00
	Mix 8	1145.00	618.00	510.00	163.00	4.10
C70	Mix 9	1125.00	633.00	475.00	166.00	9.00
	Mix10	1104.00	595.00	531.00	170.00	7.00
	Mix11	1250.00	670.00	550.00	210.00	2.00
	Mix12	1098.00	652.00	556.00	156.00	7.51
